# The regulation of healthcare professions and support workers in international context

**DOI:** 10.1186/s12960-021-00618-8

**Published:** 2021-06-08

**Authors:** Mike Saks

**Affiliations:** 1 University of Suffolk, Ipswich, UK; 2 University of Lincoln, Lincoln, UK; 3 Royal Veterinary College, University of London, London, UK; 4 University of Westminster, London, UK; 5 University of Toronto, Toronto, Canada

**Keywords:** Global North, Global South, Health professions, Health support workers, Medicine, Regulation

## Abstract

**Background:**

The objective of this paper is to outline and compare the regulation of paid healthcare professions and associated support workers in international context, bringing out the lessons to be learned as appropriate. Modern neo-liberal societies have sought to enhance healthcare through greater professional regulation, albeit in different ways and at variable pace. This general trend is illustrated with reference to medicine in the UK. However, although such reforms have helpfully cascaded to other health professions, government policy in high-income countries has not yet adequately regulated the interrelated group of non-professionalised health support workers who form the largest and least recognised part of the workforce. Nonetheless, in low- and middle-income (LMIC) countries—aside from the greater need for regulation of health professions—there is even more of an imperative to regulate the disparate, largely invisible support workforce.

**Methods:**

With reference to existing studies of the medical and wider health professions in the UK and selected other higher income societies, the importance of health professional regulation to the public is underlined in the Global North. The larger gap in the regulation of support workers in modern neo-liberal countries is also emphasised on a similar basis, with an increasingly ageing population and advances in healthcare. It is argued from the very limited patchwork of secondary literature, though, that policy-makers may want to focus even more on enhancing regulation of both the professional and non-professional workforce in LMIC societies centred mainly in the Global South, drawing on lessons from the Global North.

**Results/conclusions:**

Efforts to reform health professional regulatory approaches in more economically developed countries, while needing refinement, are likely to have had a positive effect. However, even in these societies there are still substantial shortfalls in the regulation of health support workers. There are even larger gaps in LMICs where there are fewer health professional staff and a greater dependence on support workers. With higher rates of morbidity and mortality, there is much more scope here for reforming health regulation in the public interest to extend standards and mitigate risk, following the pattern for healthcare professions in the Global North.

## Background

Enhancing health professional regulation has become a major focus of modern neo-liberal societies, in which doctors and others are typically statutorily regulated in one form or another by the state—usually through a professional body. In the UK, for example, this enhancement has involved eroding the self-regulatory authority of medical doctors as well as other players in the health professional hierarchy. As will be seen from the secondary literature sources used in methodologically constructing this paper [1], such erosion has bought with it a range of reforms which have upgraded standards and accountability. Whilst this has the potential to improve the quality of healthcare, as with parallel reforms in other societies linked to the Global North, the regulatory position of the related body of support workers—who often interface directly with health professions and form the majority of paid providers of healthcare—has been largely neglected. Support workers in healthcare are defined by delivering face-to-face care and other support of a personal or confidential nature, but are not accredited or formally regulated by a professional body [2].

The comparative neglect of support workers by government has potentially imperilled healthcare users in modern neo-liberal countries by not systematically addressing the standardisation of quality through such means as training and regulatory oversight—as the case of the UK highlights. However, despite more limited efforts to raise the standard of professional practice of the relatively smaller cadre of health professionals in LMICs, there is even more significant risk posed by the regulation of support workers. Since the regulation of the disparate and even larger numbers of support staff here is often haphazard, it is argued in this paper that, following sporadic examples of good practice by such parties as employers in the Global North, further steps need to be taken by governments and other bodies in the less economically developed world to improve quality—albeit in a manner consistent with more constrained budgets and local traditions.

## Reforming health professional regulation in modern neo-liberal countries

There has been a great change in neo-liberal health regulatory practices over the past fifty years. For example, in the UK in the late 1960s the medical profession through the General Medical Council was at the height of its self-regulatory powers, with its professional association controlling a register, internal discipline and the mainstream medical curriculum [3]. However, from the 1970s onwards it came under increasing attack, as did other professions in the Global North, in a more critical climate. This was fuelled first by the counter culture which challenged beliefs in ongoing scientific progress and endeavoured to wrest more control back to users, and subsequently by a postmodern war on expertise and the ascendance of free market ideologies [4]. This attack was exacerbated in healthcare in the UK by scandals including the medical removal of children’s organs without consent at Alder Hey Hospital and the devastating and unacceptable mortality rates arising from children’s heart surgery at Bristol Royal Infirmary [5]. The most precipitous event, however, was the murderous activities of Harold Shipman, a general practitioner who for some 30 years was found to have killed over two hundred of his patients [6].

The effect of all this was to lead a modernising government from the late 1990s in the UK to instigate the reform of the medical and other healthcare professions which was implemented in the first two decades of the twenty-first century. As a result of these reforms, key changes took place. First, the very large, overwhelmingly medically dominated, General Medical Council with over one hundred members was reduced to only 12 members, half of whom were drawn from lay people [7]. Second, complaints against doctors were no longer dealt with purely by peers in-house, but could be referred for independent adjudication, and medical professionals were to be subject to periodic revalidation by peers every 5 years to ensure their ongoing fitness to practice [8]. Moreover, an oversight body was introduced for medicine and the broader range of health professions—to whom the various reforms were cascaded—currently entitled the Professional Standards Authority for Health and Social Care. This more transparent meta-regulator is primarily funded by the professional councils for which it is responsible, and acts as a corporate rationaliser applying comprehensive standards to assess performance through review and audit [9].

Although governments still wish to increase the efficiency and effectiveness of these reforms [10], they have enhanced the service provided to patients and the wider public. Much the same might be said of other recent reforms that have taken place to improve medical regulation and governance in much of the rest of Europe, with greater emphasis on user involvement in regimes of voice, choice and co-production [11]. This is well illustrated by Scandinavia where there is also external oversight by national-level authorities responsible for licensing health professionals, which intervene in cases of malpractice or quality breaches [12]. In Australia too there have been positive federal health workforce regulatory reforms based on the National Scheme for the Registration and Accreditation of the Health Professions that has led to increased standardisation of requirements and the centralisation of governance [13]—and, as in the UK, there has been a carefully planned expansion of the scopes of practice of semi-professional groups like nurses [14]. However, the medical profession has been more resistant to encroachment on its self-regulatory powers in Canada and the United States [15].

## The position of support workers in modern neo-liberal societies

Unfortunately, though, positive reform has not been so emblematic of the steps that governments have taken in lower-level support worker regulation in their interrelated work with professionalised personnel. A systematic study carried out on behalf of the UK Departments of Health concluded in 2000 that the size of this largely invisible, non-professionalised, part of the workforce far outstripped the total numbers of medical and nursing practitioners—spanning from occupational therapy and physiotherapy aides to healthcare assistants and day care workers in over 300 different job categories [16]. It also found there was a lack of regulation by the state, other than from generic legislation in employment, health and safety, and other fields. This left much of the oversight of support workers to employers in the private and public settings such as hospitals and residential homes, as well as to individuals hiring such workers in their own homes. At times, support workers like nursing assistants and occupational therapy aides reported to professional personnel. Although there is often pre-service vetting for employment, in addition to managerial accountability and employer-sponsored development opportunities, the absence of a mandatory register exposed the public to greater risks—especially as many lacked formal qualifications.

A more recent study reaffirmed that health support workers were indeed a highly heterogenous group, but were also all too frequently poorly paid, in unstable employment and disproportionately drawn from female and ethnic minority extraction [17]. This has led some contributors—whatever trends in the deprofessionalisation of previously established health groups—to view this UK workforce as embodying the characteristics of a precariat [18]. The concept of a precariat in neo-liberal societies is based on the notion that the old-style proletariat in Marxist terms has now been replaced by a revolutionary class recruited from marginal groups with low pay and insecure working lives with the increasing privatisation of the welfare state and the spread of the New Public Management [19]. Although the sheer diversity of conditions of support workers—including those at the higher end like operating theatre practitioners who have recently professionalised—undermines the idea of this group developing a common consciousness, there is no doubt that many support workers fit into the immiserated mould of a precariat. This symbolises their less valued position in society, linked to the relative lack of regulation, and has the potential to demotivate. Their generally depressed state therefore raises questions at various levels about how far the public are best served in terms of mitigating risks [20].

The comparative lack of attention by governments to health support workers in modern neo-liberal societies has broadly been mirrored in countries like Canada, where—although provincial governments like Ontario have striven without total success to create registers and offset the low wages of the personnel involved—there are similar levels of precarity amongst this section of the healthcare workforce [21]. To be sure, in other societies like Japan, while there are still gaps in face of the most rapidly accelerating ageing population in the world, the state has done more to advance the regulation of support workers, in face of the breakdown of the traditional network of family support. Here it has identified centrally defined new qualifications for Certified Care Workers and Home Visiting Care Workers, who operate alongside the lesser qualified but long-standing *Tsukisoifu* in providing healthcare support [22]. Indeed, in a number of countries such as Denmark users themselves have been elevated up the health pecking order to the level of peer support workers in mental health and other settings based on their experiential knowledge and further training. This highlights another form of credentialing in the regulatory framework of non-professionalised workers in tending to the needs of clients and their informal carers [23].

## Reforming the health professions in low- and middle-income countries

To return to the health professions per se, in the economically developing world, governments are faced with quite different, but also diverse, circumstances. Health is a human right and enhancing it is a central part of the United Nations’ Sustainable Development Goals which apply globally [24]. Nonetheless, with a typically lower gross domestic product and associated higher mortality and morbidity rates, there are even greater health challenges [25]—to add to the frequently arising emergencies and humanitarian crises in this sector, as well as sometimes unhelpful colonial legacies [26]. However, one of the difficulties that is faced in much of the Global South, is that there are far fewer qualified health professionals available compared to the population served. As a key indicator, the number of doctors per one thousand inhabitants in modern neo-liberal societies spans from 1.5 in Singapore to 3.7 in The Netherlands [27]. World Health Organization (WHO) data, though, shows that over 40 per cent of member states have less than one doctor per one thousand inhabitants and 26 per cent have less than 0.3 [28]. Moreover, this shortfall is not necessarily counterbalanced by the existence of other health professionals in LMICs. The latest WHO figures, for instance, show that of over 20 million nurses and midwives worldwide, half of the member states have less than three such professionals per one thousand population and 25 per cent have less than one [29].

Nonetheless, some countries like Cambodia and Vietnam have made remarkable progress with the support of international partners in developing the regulatory framework for nursing and other health professionals, underpinned by legislation [30]. The challenge of regulating health professions in other such societies has been met by providing additional educational infrastructure. This can be illustrated in Brazil by the recent federal government requirement that doctors undertake substantial additional training if they are to practise recognised forms of complementary and alternative medicine [31]—as well as the dictum that non-specialised nurses need to be developed further to fulfil wider roles in healthcare [32]. Although COVID-19 has interrupted quality improvement for already qualified healthcare professionals in LMICs, as in their modern neo-liberal counterparts, there have been ways to maintain this through distance learning and other methods despite their usually more limited technological infrastructure [33]. At the other end of the spectrum, delicate balances have also had to be reached in pursuing such initiatives as increasing the number of rural physicians in the Asia-Pacific region and elsewhere without diminishing the quality of graduating doctors [34] and in face of pressures of international mobility of healthcare graduates to more affluent countries [35].

However, it is important to see beyond more explicit regulatory requirements. In India, for example, medical and other health services are formally regulated through national and state health boards by developing standards, protocols, norms and guidelines—as well as through the accreditation of facilities. This sits alongside the direct regulation of doctors as a profession through the Medical Council of India which oversees a register alongside ethical, training and practice standards. However, no matter how well-intentioned such measures are following the colonial legacy of the UK, there remain many regulatory issues that have not yet been satisfactorily addressed in India such as non-compliance; lack of enforcement; bribery, corruption and overcharging; and a failure to assess the enforcement activities of the regulators in both education and practice [36]. These and other concerns seem to have led to a breakdown of trust at all levels between patients, providers and the regulators. This is replicated in a number of other LMICs where there are doubts about the competency of professions such as doctors and nurses [37], the enforcement of continuing professional development [38], and the provision of supportive supervision in building capacity, improving the quality of care and enhancing clinical outcomes [39]. This is exacerbated by the influence of market forces, including on the proliferation of low-quality private schools for health professionals in LMICs, which mean that regulations do not achieve their intended effect [40].

## The position of support workers in low- and middle-income countries

Unfortunately, it is not only the number of doctors that is disproportionally spread between modern neo-liberal and LMICs. As the WHO has noted, countries with the highest comparative health needs and the greatest burden of disease have the lowest number of non-professional health workers. Thus, for example, African countries with less than one per cent of the world’s financial resources, bear some 22 per cent of the global burden of disease, but their populations have access to only 3 per cent of health workers [28]. This makes such societies even more dependent on the smaller proportion of support workers who play a potentially more impactful role than in more economically developed countries. As such, for all the shortfalls in their regulation in the more affluent Global North, it is even more imperative that they are stringently regulated in the Global South in delivering vital services to a wider public, including those in less populated and impoverished areas—from orchestrating mass public health education initiatives to carrying out large-scale vaccination programmes.

Examples of lesser trained groups of support workers include those like community health workers operating as part of primary healthcare teams in post-apartheid South Africa who—following a model from Brazil—visit households and communities to screen for diseases and risk factors and educate on basic health issues [41]. Needless to say, groups such as these and yet to be qualified students have proved invaluable more widely in LMICs in combatting the recent COVID-19 pandemic, as in some more wealthy countries. This model for students was certainly extant in India, where the motivation and experience of community health workers has also potentially enabled them to become WHO-designated One Health Activists [42]. In Thailand too non-professional paid care givers recruited from village health volunteers are trained to provide personal assistance and health services to the elderly in their community—with technical support from a multidisciplinary health team of doctors, nurses, and physiotherapists [43]. Positive as these developments are, there remain reservations. These are highlighted in Kenya where there have been warnings about expanding the number of health care assistants to address nursing deficits because of the need to first define scopes of practice and develop an appropriate skill mix, as was learnt from the experience of the European Union [44].

It is also apparent that the predominantly female part-time community health workers in India like many support workers in countries from Bangladesh and Nepal to Iran and Ethiopia are even more like a precariat than their counterparts in Europe and North America—as they have more problematic working conditions and extremely low and irregular payments, with all the implications that this carries for their incentivisation and motivation in completing tasks alongside household responsibilities [45]. For such workers there are also more explicit regulatory issues that have been raised concerning infrequent supervision and ongoing training. A recent study in Eswatini in sub-Saharan Africa, for example, which is facing one of the severest shortages of human resources for health, highlighted the extreme dilemmas faced in the accreditation and regulation of support work. On the one hand, the task-shifting part played by lay health workers was welcomed in such roles as administering intramuscular injections as this improved medication adherence, reduced stigma and removed some transport-related access barriers to treatment. On the other, key stakeholders—including representatives from the Ministry of Health, professional regulatory institutions, and academia—were fearful of poor standards of care leading to adverse events like overdosing and infections. This was because of the inadequacy of training, supervision and more general regulatory support, and the absence of a standardised curriculum [46].

## Conclusions

In analysing the interrelated position of health professions and support workers globally, this paper has highlighted how health professional regulation itself has been enhanced further in the modern neo-liberal world, with potentially positive results for users and the wider public. However, ongoing improvements here are still necessary. This is an even more pressing requirement for health professions in the less-well served LMICs with significantly higher mortality and morbidity rates. From the illustrative studies presented here, the main lesson is that standards of healthcare professions are raised and applied more consistently as they have been in the Global North, together with their numbers within more limited national budgets.

In both the Global North and South, though, the development of regulation of the ever more crucial, yet largely invisible, non-professionalised support worker labour force is even more imperative—albeit in different ways and for differing reasons. In meeting this need, which is much more pressing in the LMICs, the culture and traditions of countries in the Global South should not be insensitively overridden. As recent research has shown, support workers like the South African community health workers, may not have the same educational levels as their equivalents in more affluent societies, but commendably have an understanding of their own communities and can therefore deliver culturally resonant health services [41]. Indeed, their areas of success lead us to question whether some less highly flung health professions and support workers in the Global North are using their far more extensive training to best effect in the healthcare arena.

Nonetheless, given the fragmentary evidence currently available—especially in LMICs—more rigorous, sensitive and extensive research is needed on the regulation of the health practitioner workforce globally, not least because of the methodological bear pits involved in comparative health research [47]. Future research also needs to look at the detail of the relationship between health professionals and support workers since this is critical to the delivery of high-quality services. In more economically developed countries this is particularly crucial in relation to allied health professions like physiotherapists and podiatrists who have a closer day-to-day relationship with health support workers than doctors, despite the typically directional role of the latter [48]. While government policy-makers in LMICs are still dealing with the question of how best to cope with quality control of the ever-expanding cohorts of support workers, closing the gap between the regulatory practices in the Global North and Global South may lie partly in greater cooperation and coordination beyond the single nation state—whether through the sharing of good practice on the ground or the proactive pan-national leadership of bodies like the WHO [49]. In addition, the role of private national and international corporate bodies in improving global regulatory practices should not be ignored—even if the current impact of such players as pharmaceutical companies has been far from exemplary, not least in economically developing societies [50].

## Data Availability

Data are publically available and key sources are cited.
